# CE-MS-Based Identification of Uremic Solutes Specific to Hemodialysis Patients

**DOI:** 10.3390/toxins13050324

**Published:** 2021-04-30

**Authors:** Yasutoshi Akiyama, Koichi Kikuchi, Takafumi Toyohara, Eikan Mishima, Chitose Suzuki, Takehiro Suzuki, Masaaki Nakayama, Yoshihisa Tomioka, Tomoyoshi Soga, Takaaki Abe

**Affiliations:** 1 Laboratory of Oncology, Pharmacy Practice and Sciences, Graduate School of Pharmaceutical Sciences, Tohoku University, Sendai 980-8578, Japan; yasutoshi.akiyama.b4@tohoku.ac.jp (Y.A.); yoshihisa.tomioka.a6@tohoku.ac.jp (Y.T.); 2 Division of Nephrology, Endocrinology and Vascular Medicine, Tohoku University Graduate School of Medicine, Sendai 980-8574, Japan; koichikikuchi@med.tohoku.ac.jp (K.K.); toyohara@med.tohoku.ac.jp (T.T.); eikan@med.tohoku.ac.jp (E.M.); chitose@med.tohoku.ac.jp (C.S.); suzuki2i@med.tohoku.ac.jp (T.S.); 3 Division of Medical Science, Tohoku University Graduate School of Biomedical Engineering, Sendai 980-8574, Japan; 4 Department of Nephrology, St. Luke’s International Hospital, Tokyo 104-8560, Japan; nakayama@luke.ac.jp; 5 Institute for Advanced Biosciences, Keio University, Tsuruoka 997-0052, Japan; soga@sfc.keio.ac.jp; 6 Department of Clinical Biology and Hormonal Regulation, Tohoku University Graduate School of Medicine, Sendai 980-8574, Japan

**Keywords:** uremic solutes, hemodialysis, chronic kidney disease, CE-MS

## Abstract

Uremic toxins are suggested to be involved in the pathophysiology of hemodialysis (HD) patients. However, the profile of uremic solutes in HD patients has not been fully elucidated. In this study using capillary electrophoresis mass spectrometry (CE-MS), we comprehensively quantified the serum concentrations of 122 ionic solutes before and after HD in 11 patients. In addition, we compared the results with those in non-HD patients with chronic kidney disease (CKD) to identify HD patient-specific solutes. We identified 38 solutes whose concentrations were higher in pre-HD than in CKD stage G5. Ten solutes among them did not significantly accumulate in non-HD CKD patients, suggesting that these solutes accumulate specifically in HD patients. We also identified 23 solutes whose concentrations were lower in both pre- and post-HD than in CKD stage G5. The serum levels of 14 solutes among them were not affected by renal function in non-HD patients, suggesting that these solutes tend to be lost specifically in HD patients. Our data demonstrate that HD patients have a markedly different profile of serum uremic solute levels compared to that in non-HD CKD patients. The solutes identified in our study may contribute to the pathophysiology of HD patients.

## 1. Introduction

Chronic kidney disease (CKD) is now recognized as a worldwide public health problem [1]. In CKD patients, various uremic solutes, which are normally excreted into urine by the kidney, accumulate as the renal function declines. If these retained solutes have a harmful effect on the body, they are called “uremic toxins”. As uremic toxins can cause various symptoms and increase mortality [2], clarifying the profile of uremic solutes is urgently needed for better outcomes in CKD patients. With the progress of identification technology such as mass spectrometry (MS) and nuclear magnetic resonance (NMR), more than 100 organic solutes have been identified and reported as uremic solutes or uremic toxins [3,4,5,6,7]. We also reported a comprehensive profile of uremic solutes in non-HD CKD patients using capillary electrophoresis mass spectrometry (CE-MS) [8].

When CKD progresses to end-stage renal disease (ESRD), most patients will require renal replacement therapy (RRT) to maintain homeostasis of the internal environment. HD is the most common modality worldwide among RRTs [9,10]. It is well known that HD patients suffer from several specific symptoms such as disturbances of the immune system, accelerated atherosclerosis and malnutrition. These symptoms are now called malnutrition–inflammation–atherosclerosis (MIA) syndrome [11] or residual syndrome [12]. It is believed that such symptoms are at least partly due to the uremic toxins that are poorly removed by HD. Although there have been several reports identifying the uremic solutes that accumulate in HD patients [4,13,14,15], these data are based on comparisons between HD patients and a healthy population with normal renal function. Therefore, the profile of the uremic solutes in HD patients is still unclear, especially in terms of the specific characteristics compared to non-HD CKD patients. In addition, although the primary aim of HD is to remove uremic solutes, the efficiency of removal by HD remains largely unknown for most uremic solutes except small molecules such as urea and creatinine [16]. Thus, a comparison of the profile of uremic solutes between HD patients and non-HD CKD patients would be a valuable approach for the development of more efficient dialyzers as well as for clarification of the pathophysiology of HD patients. 

Here we comprehensively quantified the serum concentrations of 122 solutes (54 anions and 68 cations) both before and after HD in 11 patients by CE-MS. In order to accurately compare the values between HD patients and non-HD CKD patients, CE-MS was performed under the same conditions as in non-HD CKD patients, as previously reported [8]. We show that HD patients have a different profile of uremic solutes by identifying molecules that specifically increased or decreased in HD patients. In addition, we demonstrate the profile of removal of these solutes by HD through calculation of the removal rate.

## 2. Results

### 2.1. Baseline Characteristics of HD Patients and Non-HD CKD Patients

The characteristics of the HD patients are shown in Table 1. The sample group consisted of six females and five males. All the patients had no residual renal function. The mean age was about 62 years old and the mean duration of HD treatment was about 8 years.

As we previously reported [8], non-HD CKD patients consisted of 41 patients whose estimated glomerular filtration rate (eGFR) was below 60 mL/min/1.73 m^2^. The numbers of patients in CKD stage G3 (30 ≤ eGFR <60), G4 (15–30) and G5 (<15) were 11, 17 and 13, respectively. The characteristics of CKD stage G5 patients are also shown in Table 1.

### 2.2. Solutes Whose Serum Levels Were Higher in HD Patients Than in Non-HD CKD Patients

**Figure 1:**
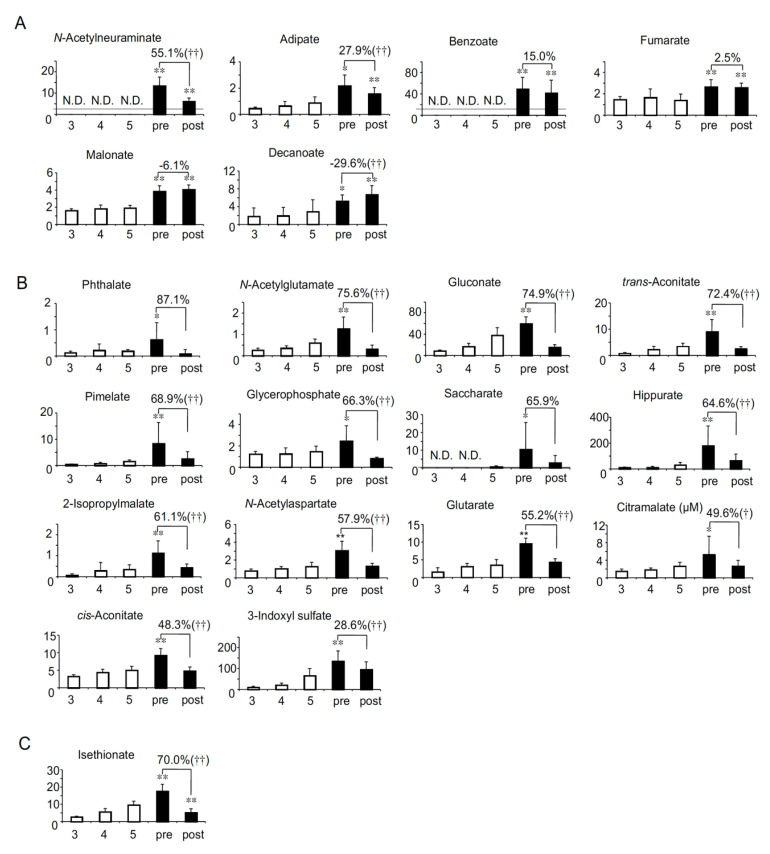
The 21 anionic solutes whose concentrations (µM) in pre-HD were significantly higher than in chronic kidney disease (CKD) stage G5. (**A**) The six solutes whose levels were higher in both pre- and post-HD than in CKD stage G5 by HD. (**B**) The 14 solutes that were removed to the same levels of CKD stage G5 by HD. (**C**) The one solute that was removed to below the levels of CKD stage G5 by HD. Percent values indicate removal rates. N.D.: not detected. CKD stage G3, stage G4, stage G5, pre-HD and post-HD are abbreviated as 3, 4, 5, pre and post, respectively. † *p* < 0.05 between pre- and post-HD, †† *p* < 0.01 between pre- and post-HD. * *p* < 0.05 vs. CKD stage G5, ** *p* < 0.01 vs. CKD stage G5. Gray line represents the detection limit.

**Figure 2:**
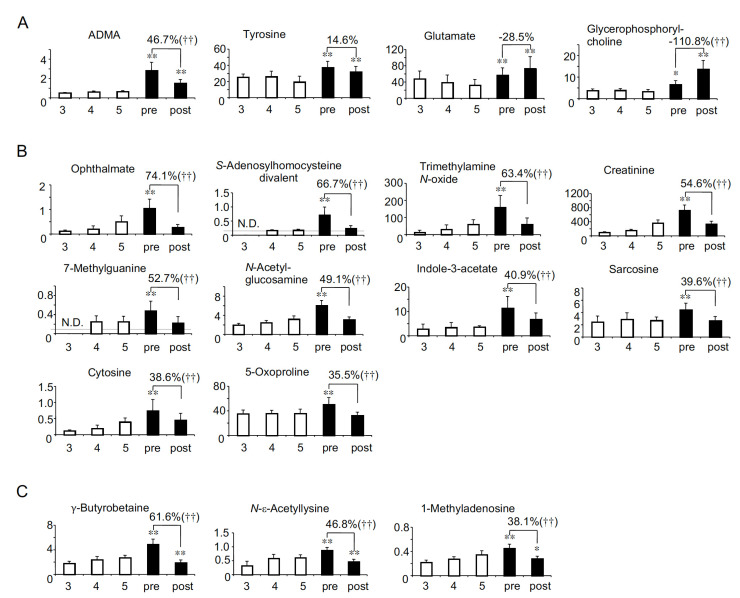
The 17 cationic solutes whose concentrations (µM) in pre-HD were significantly higher than in CKD stage G5. (**A**) The four solutes whose levels were higher in both pre- and post-HD than in CKD stage G5. (**B**) The 10 solutes that were removed to the same levels of CKD stage G5 by HD. (**C**) The three solutes that were removed to below the levels of CKD stage G5 by HD. ADMA: asymmetric dimethylarginine. Percent values indicate removal rates. N.D.: not detected. CKD stage G3, stage G4, stage G5, pre-HD and post-HD are abbreviated as 3, 4, 5, pre and post, respectively. †† *p* < 0.01 between pre- and post-HD. * *p* < 0.05 vs. CKD stage G5, ** *p* < 0.01 vs. CKD stage G5. Gray line represents the detection limit.

As uremic solutes accumulate with the decline of renal function in non-HD CKD patients, their concentrations are estimated to be highest in CKD stage G5. In contrast, in HD patients, they are generally estimated to be highest just before HD because HD is the only way to remove these solutes. In order to profile the uremic solutes specific to HD patients, we first compared the serum levels between non-HD CKD stage G5 and before HD (pre-HD). Among 122 solutes, we identified 21 anionic solutes (Figure 1) and 17 cationic solutes (Figure 2) whose concentrations in pre-HD were significantly higher than in non-HD CKD stage G5. Most solutes (14 anions and 10 cations) were decreased to the same level as CKD stage G5 (Figure 1B and Figure 2B). As shown in Figure 1C and Figure 2C, one anion and three cations were decreased to below the level of CKD stage G5. In contrast, the serum concentrations of six anions (N-acetylneuraminate, adipate, benzoate, fumarate, malonate and decanoate) and four cations (ADMA, tyrosine, glutamate and glycerophosphorylcholine) were significantly higher in both pre- and post-HD than in CKD stage G5 (Figure 1A and Figure 2A), suggesting that HD patients suffer from higher levels of these 10 solutes at all times compared to non-HD CKD patients.

### 2.3. Solutes the Serum Levels of Which Were Lower in HD Patients Than in Non-HD CKD Patients

**Figure 3:**
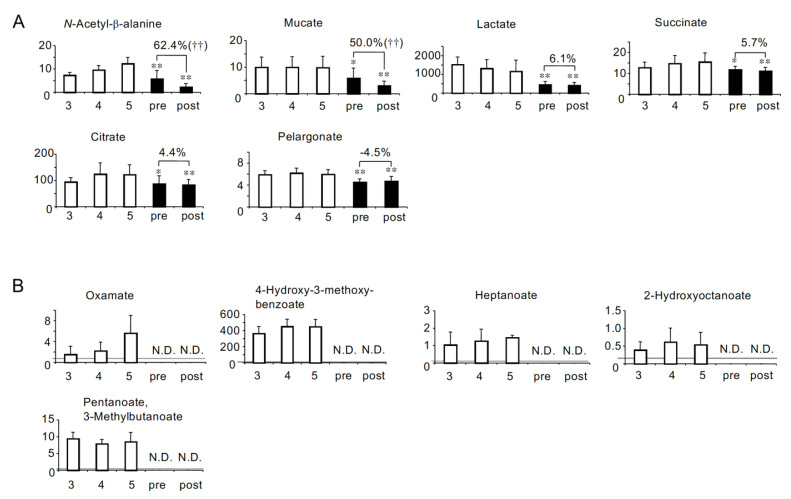
The 11 anionic solutes whose concentrations (µM) were lower in HD patients than in CKD stage G5. (**A**) The six solutes whose concentrations were significantly lower in both pre- and post-HD than in CKD stage G5. (**B**) The five solutes that were not detected in HD patients. Percent values indicate removal rates. N.D.: not detected. CKD stage G3, stage G4, stage G5, pre-HD and post-HD are abbreviated as 3, 4, 5, pre and post, respectively. †† *p* < 0.01 between pre- and post-HD. * *p* < 0.05 vs. CKD stage G5, ** *p* < 0.01 vs. CKD stage G5. Gray line represents the detection limit.

**Figure 4:**
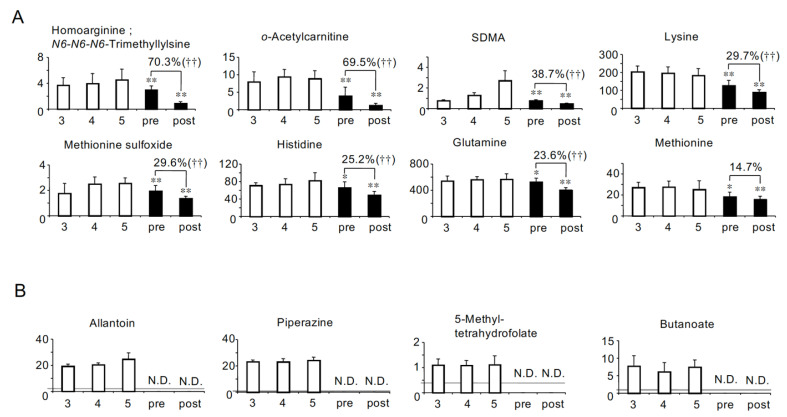
The 12 cationic solutes whose concentrations (µM) were lower in HD patients than in CKD stage G5. (**A**) The eight solutes whose concentrations were significantly lower in both pre- and post-HD than in CKD stage G5. (**B**) The four solutes that were not detected in HD patients. SDMA: symmetric dimethylarginine. Percent values indicate removal rates. N.D.: not detected. CKD stage G3, stage G4, stage G5, pre-HD and post-HD are abbreviated as 3, 4, 5, pre and post, respectively. †† *p* < 0.01 between pre- and post-HD. * *p* < 0.05 vs. CKD stage G5, ** *p* < 0.01 vs. CKD stage G5. Gray line represents the detection limit.

We hypothesized that the symptoms of HD patients could be due to the shortage of beneficial metabolites as well as the accumulation of uremic toxins. Therefore, we next identified the solutes whose concentrations were significantly lower in both pre- and post-HD than in CKD stage G5. As a result, six anionic (Figure 3A) and eight cationic solutes (Figure 4A) were identified. In addition, five anions (oxamate, 4-hydroxy-3-methoxybenzoate, heptanoate, 2-hydroxyoctanoate and pentanoate/3-methylbutanoate) and four cations (allantoin, piperazine, 5-methyltetrahydrofolate and butanoate) were not detected in HD patients (Figure 3B and Figure 4B).

### 2.4. Profiling of HD Patient-Specific Uremic Solutes

To clarify the differences between HD patients and non-HD CKD patients more clearly, we classified these solutes into six groups based on two characteristics: (1) their correlations with eGFR in non-HD CKD patients [8] and (2) whether they accumulated or decreased in HD patients compared to non-HD CKD patients with stage G5 (Table 2). The solutes whose concentrations were higher in HD patients than non-HD CKD stage G5 patients were classified into groups A–C. It is suggested that HD patients suffer from higher concentrations of group A–C solutes compared to non-HD CKD patients. Group A is defined as solutes that were accumulated in both HD patients and non-HD CKD patients. Twenty-six solutes were classified into group A. These solutes can be called “typical uremic solutes” because they accumulate as eGFR declines in non-HD CKD patients. Group B is defined as solutes that did not significantly accumulate with the decline of eGFR in non-HD CKD patients. Ten solutes were classified into group B. It is suggested that the group B solutes may tend to accumulate specifically in HD patients. Therefore, they could be called “HD patient-specific uremic solutes”. Tyrosine and glutamate were classified into group C because their serum levels were positively correlated with eGFR in non-HD CKD patients [8,17], which suggested different metabolic profiles between HD patients and non-HD CKD patients. 

On the other hand, the solutes whose concentrations were lower in HD patients than non-HD CKD stage G5 patients were classified into groups D–F. Similar to Group A, Group D can be called “typical uremic solutes” because Group D solutes accumulate as eGFR declines in non-HD CKD patients. Eight solutes were classified into group D. It is suggested that the accumulation of the group D solutes was less significant in HD patients than in non-HD CKD patients. Group E is defined as solutes that did not significantly accumulate with the decline of eGFR in non-HD CKD patients. Fourteen solutes were classified into group E. It is suggested that HD patients may have had a tendency to lose these solutes. Lactate was classified into group F because of the positive correlation of its serum level with eGFR. It is suggested that lactate is decreased in HD patients more significantly than in non-HD CKD patients.

### 2.5. Removal Rate

As the profile of the removal efficiency of uremic solutes by HD will be valuable for developing better dialyzers as well as for the better understanding of the pathophysiology of HD patients, we calculated the removal rate of all the solutes. Although most solutes were significantly removed by HD, 22 anions (benzoate, fumarate, malonate, phthalate and saccharate in Figure 1, lactate, succinate, citrate and pelargonate in Figure 3, and quinate, pyruvate, malate, hexanoate, azelate, sebacate, cysteine S-sulfate, 2-oxoglutarate, octanoate, glycolate, 2-oxoisopentanoate, 2-hydroxyisobutyrate/2-hydroxybutyrate and 3-hydroxybutyrate in Figure S1) and 16 cations (tyrosine in Figure 2, methionine in Figure 4, and 1-methyl-2-pyrrolidone, cysteine-glutathione disulphide, ethanolamine phosphate, creatine, β-alanine, isoleucine, phenylalanine, leucine, 1-methylnicotinamide, aspartate, urocanate, hypoxanthine, guanosine and inosine in Figure S2) were not significantly removed. Furthermore, the serum concentrations of three anions (decanoate in Figure 1A, and laurate and 4-methyl-2-oxopentanoate in Figure S1) and two cations (glycerophosphorylcholine in Figure 2A, and tryptophan in Figure S2) were significantly elevated after HD.

## 3. Discussion

The principal aim of this study was to identify the uremic solutes specifically involved in the pathophysiology in HD patients. Here we comprehensively quantified the serum concentrations of various solutes in HD patients and compared them with those in non-HD CKD patients. We summarized the results of this study in Table 2. We first identified 38 solutes (21 anions and 17 cations) whose concentrations were significantly higher in pre-HD than CKD stage G5 (Figure 1 and Figure 2 and groups A-C in Table 2). These solutes may be involved with the pathophysiology in HD patients, since these solutes accumulated at higher levels in HD patients, at least temporarily, than in non-HD CKD patients. Generally, uremic solutes are defined as solutes that accumulate as renal function declines. Therefore, whether the solute is “uremic” is determined based on the characteristics in non-HD CKD patients. For this reason, 26 solutes in group A and 8 solutes in Group D can be called “typical uremic solutes”, since they accumulate with the decline of eGFR in non-HD CKD patients. In contrast, we also identified the solutes that can accumulate only in HD patients (Table 2-Group B) in this study. The serum concentrations of the group B solutes did not show a correlation with eGFR in non-HD patients in our previous study [8]. Particularly, N-acetylneuraminate and benzoate (Figure 1A) were not detected in non-HD CKD patients even in stage G5. Therefore, they could be called “HD patient-specific uremic solutes”. On the other hand, the serum concentrations of two amino acids, tyrosine and glutamate, showed a positive correlation with eGFR (Table 2-Group C). These serum levels seemed to be recovered to near the levels in CKD stage G3 (Figure 2A), the significance of which remains unclear. Further study is needed to clarify the metabolic significance of the differences between HD patients and non-HD CKD patients.

It should be noted that the biological functions and/or toxicities are still unknown for most of the uremic solutes evaluated in this study. However, various toxic effects have been reported for at least five solutes (hippurate, and 3-indoxyl sulfate in Figure 1B, and ADMA in Figure 2A, and trimethylamine *N*-oxide and indole-3-acetate in Figure 2B) and they have been classified as uremic toxins [18,19,20,21]. In addition, we reported that *trans*-aconitate (Figure 1B) is accumulated with the decline of renal function and shows the toxicity in vitro and in vivo [22]. It is suggested that HD patients may suffer from these toxins more strongly than patients with CKD stage G5.

We also identified 23 solutes whose concentrations were lower in both pre- and post-HD than CKD stage G5 (Figure 3 and Figure 4). Similar to the solutes that accumulated more significantly in HD patients, we classified these solutes into three groups by the correlation with eGFR (Groups D-F in Table 2). Eight solutes classified into group D could be called “uremic solutes that accumulate less significantly in HD patients”. Among them, symmetric dimethylarginine (SDMA) has been reported to cause vascular damage by increasing reactive oxygen species (ROS) production in monocytes [23]. It is suggested that the toxicity of SDMA may be reduced in HD patients compared with patients in CKD stage G5. On the other hand, we also noted the negative effects of the shortage of metabolic solutes. Fourteen solutes classified into group E could be called “solutes specifically lost in HD patients”, because their serum concentrations did not significantly change with the decline of eGFR in non-HD CKD patients. In addition, lactate, which was classified into group F, could be called “a solute that decreased more significantly in HD patients”. The lack of these solutes may be involved in the symptoms in HD patients. For example, 5-methyltetrahydrofolate (Figure 4B) is known to be the major form of folate circulating in the serum and to be involved in DNA synthesis and maintenance [24], and it was not detected in HD patients (Figure 4B). Although it has been reported that a decrease in the serum concentrations of folate does not necessarily indicate a folate deficiency [25], it can be said that HD patients in our study had a tendency toward a lack of folate. In addition, butanoate, also called butyrate, was not detected in either pre- or post-HD (Figure 4B and Table 2-Group E). Butanoate is a saturated fatty acid containing four carbon molecules and known to be one of the short-chain fatty acids (SCFAs). SCFAs are produced by colonic microbiota during the fermentation of nondigestible polysaccharides. It has been reported that butanoate is a major energy source for colonocytes [26]. Since it has been reported that HD patients showed decreases in colonic microbial families possessing butanoate-forming enzymes [27], the decrease in the levels of butanoate in HD patients in our study may have been caused by changes in the colonic microbiota. In our study, we also evaluated the concentrations of other SCFAs, pentanoate/3-methylbutanoate (pentanoate is an SCFA containing five carbon molecules) and hexanoate (SCFA containing six carbon molecules). Although the serum concentrations of hexanoate were detected in both pre- and post-HD and at the same level as those in CKD stage G5 (Figure S1), pentanoate/3-methylbutanoate (Figure 3B) was not detected in HD patients similarly to butanoate. In addition, heptanoate, which is an SCFA containing seven carbon molecules, was not detected in HD patients either (Figure 3B). There is accumulating evidence that SCFAs have various effects on the maintenance of cellular homeostasis such as anti-inflammatory, anti-tumorigenic and anti-microbial effects [28]. These data suggest that HD patients may suffer from negative effects due to the lack of SCFAs.

In this study, we showed the removal rate of each solute by HD. The removal rates ranged widely from 87.1% (for phthalate in Figure 1B) to −260.1% (for 3-hydroxybutyrate in Figure S1). Because the molecular weight of all the solutes analyzed in our study was <500 Da (from 75.0 to 460.2 *m*/*z*), the solutes can theoretically pass through the dialyzer membrane and be removed from the blood if they exist in free form. Therefore, to some extent, these data should reflect the tendency of each solute to bind to serum proteins such as albumin. For instance, guanidinosuccinate (Figure S2) has been classified as a small water-soluble (i.e., non-protein-bound) uremic toxin [7], and its removal rate was relatively high (76.7%). On the other hand, the removal rate for 3-indoxyl sulfate (Figure 1B), which has been classified as a protein-bound uremic toxin, was relatively low (28.6%). Interestingly, the serum concentrations of three anions (decanoate in Figure 1A, laurate and 4-methyl-2-oxopentanoate in Figure S1) and two cations (glycerophosphorylcholine in Figure 2A and tryptophan in Figure S2) were significantly elevated after HD. These increases may be explained by the distribution of solutes from tissue to blood during HD. For example, it has been reported that the blood glutamate concentration was increased during HD [29]. The authors reported that the blood glutamate levels changed dynamically during HD, i.e., decreased in the first 3 h and increased at the fourth hour. They postulated that glutamate may have been released from neurons in order to compensate for the osmotic disequilibrium caused by the removal of urea during HD [29]. Although not statistically significant, the serum glutamate levels showed a tendency to increase after HD in our study (removal rate: −28.5%; Figure 2). In addition, it has been reported that the plasma ADMA concentration increased 1 h after HD compared to before HD, and decreased significantly 5 h after HD compared to 1 h after HD [30]. The authors speculated that the increase in the ADMA levels after HD could be partly the result of a redistribution of tissue ADMA into the plasma compartment during HD [30]. Similarly, there could be a possibility that the distribution from the tissue into the serum compartment during HD affected the removal rates analyzed in this study. One method to evaluate the removal efficiency of HD more accurately is to calculate removal amounts by analyzing the dyalisate concentrations of solutes. However, dyalisate was not examined in our analysis, which is one of the limitations of this study. Further investigations are needed to fully elucidate the removal profiles of uremic solutes by HD.

Another limitation of this study is the small number (n = 11) of patients. However, concentrations of many solutes showed relatively small standard deviations sufficient to yield statistical significance, which suggests that the serum concentrations of many solutes in this study are largely affected by the condition of HD therapy rather than other factors such as duration of HD, age, sex and so on. On the other hand, the solutes with relatively higher standard deviation (e.g., pyruvate and 3-hydroxybutyrate in Figure S1, and inosine in Figure S2) may be more strongly affected by factors other than the condition of HD.

## 4. Conclusions

In summary, we identified 38 solutes and 23 solutes whose serum concentrations in HD patients were higher and lower than in non-HD CKD patients, respectively. Among them, the accumulation of 10 solutes and the loss of 14 solutes were specific to HD patients compared to non-HD CKD patients. We clearly showed that HD patients had significantly different profiles of uremic solutes compared to non-HD CKD patients. The accumulation or shortage of these solutes may be involved in the symptoms in HD patients. Our data provide valuable information for a better understanding of the pathophysiology in HD patients and the profile of uremic solute removal by HD. In addition, our data should also provide a clue for identifying therapeutic target solutes in HD patients and for developing more efficient dialyzers.

## 5. Materials and Methods

### 5.1. Study Population

The study population consisted of 11 CKD patients who were treated using standard HD, with three sessions/week (4–5 h/session), using high-performance biocompatible dialyzer with polysulfone membrane. All patients had no residual renal function. Serum samples were collected just before and after HD session and subjected to CE-MS analysis.

The study protocol complied with the Declaration of Helsinki and was approved by the Committees on the Ethics of Human Research of Tohoku University and informed consent was obtained from each participant. 

Serum concentrations of solutes in non-HD CKD patients were obtained by reanalysis of our previous study with 41 CKD patients [8]. Briefly, the population of the study consisted of 41 patients whose estimated glomerular filtration rate (eGFR) was below 60 mL/min/1.73 m^2^. The numbers of patients in CKD stage G3 (30 ≤ eGFR <60), G4 (15–30) and G5 (<15) were 11, 17 and 13, respectively. We showed the mean serum concentrations of solutes in each CKD stage and the correlation between serum concentrations and eGFR evaluated in our previous study [8] in Tables S1 and S2.

### 5.2. CE-MS Measurement for Metabolome Analysis

Fifty microliters of the serum was immediately plunged into 450 μL of methanol containing 20 μM each of methionine sulfone (Wako, Japan), MES (Dojindo, Japan) and CSA (D-Camphol-10-sulfonic acid, Wako, Japan) as internal standards. Two hundred microliters of deionized water and 500 µL of chloroform were then added, and the mixture was thoroughly mixed. The solution was centrifuged at 4600× *g* for 5 min at 4 °C, and the upper aqueous layer was centrifugally filtered through a Millipore 5-kDa cut-off filter (Millipore, Burlington, MA, USA) to remove proteins. The filtrate was lyophilized and dissolved in 50 μL of Milli-Q water containing 200 μL each of 3-aminopyrrolidine (Sigma-Aldrich, St. Louis, MO, USA) and trimesate (Wako, Japan) as reference compounds.

All CE-TOFMS experiments were performed using the Agilent CE capillary electrophoresis system (Agilent Technologies, Waldbronn, Germany), the Agilent G3250AA LC/MSD TOF system (Agilent Technologies, Santa Clara, CA, USA), the Agilent 1100 series binary HPLC pump, the G1603A Agilent CE-MS adaptor and the G1607A Agilent CE-ESI-MS sprayer kit. G2201AA Agilent ChemStation software for CE and the Analyst QS for Agilent TOFMS software were used for system control and data acquisition.

We performed a comprehensive and quantitative analysis of charged metabolites by CE-MS as described previously [8,31,32]. Briefly, to analyze cationic compounds, a fused silica capillary (50 µm i.d. × 100 cm) was used with 1 M formic acid as the electrolyte [31]. Approximately 3 nL of sample solution was injected at 50 mbar for 3 s and 30 kV of voltage was applied. The capillary temperature was maintained at 20 °C and the sample tray was cooled below 5 °C. Methanol/water (50% *v*/*v*) containing 0.1 µM hexakis (2,2-difluoroethoxy) phosphazene was delivered as the sheath liquid at 10 µL/min. ESI-TOFMS was performed in positive ion mode, and the capillary voltage was set to 4 kV. Automatic recalibration of each acquired spectrum was achieved using the masses of the reference standards ([13 C isotopic ion of a protonated methanol dimer (2 MeOH + H)]^+^, *m*/*z* 66.0632) and ([hexakis(2,2-difluoroethoxy)phosphazene + H]^+^, m/z 622.0290). To identify metabolites, relative migration times of all peaks were calculated by normalization to the reference compound 3-aminopyrrolidine. The metabolites were identified by comparing their *m*/*z* values and relative migration times to the metabolite standards. Quantification was performed by comparing peak areas to calibration curves generated using internal standardization techniques with methionine sulfone. The other conditions were identical to those described previously [33].

To analyze anionic metabolites, a commercially available COSMO(+) (chemically coated with cationic polymer) capillary (50 µm i.d. × 105 cm) (Nacalai Tesque, Japan) was used with a 50 mM ammonium acetate solution (pH 8.5) as the electrolyte. Sample solution (30 nL) was injected at 50 mbar for 30 s and −30 kV of voltage was applied. Methanol/5 mM ammonium acetate (50% *v*/*v*) containing 0.1 µM hexakis (2,2-difluoroethoxy) phosphazene was delivered as the sheath liquid at 10 µL/min. ESI-TOFMS was performed in negative ion mode, and the capillary voltage was set to 3.5 kV. For anion analysis, CSA was used as the reference and the internal standards, respectively. The other conditions were identical to those described for cationic metabolite analysis.

Although HD samples were not measured together with non-HD CKD samples, we used methionine sulfone as an internal standard to correct for changes in sensitivity of the mass spectrometry.

### 5.3. Statistical Analyses

The data were expressed as means ± SD. Samples below the detection limit were assigned the value of the detection limit and subjected to statistical analysis. However, if the values were below the detection limit in all the samples in a group, the result was noted as N.D. (not detected). All the data of serum concentrations (mean ± SD) are shown in Tables S1 and S2. 

The removal rate was calculated according to the following formula: 1- (mean post-HD value/mean pre-HD value) × 100 (%). 

For statistical analysis, Dunnett’s multiple comparison test was performed to compare values in CKD stage G5 versus those in pre-HD or post-HD. Paired t-test was used to compare values between pre-HD and post-HD. Benjamini–Hochberg procedure was used for correction for multiple hypothesis testing. A false discovery rate (FDR) < 0.05 was considered to be statistically significant.

## Figures and Tables

**Table 1:** Baseline characteristics of HD patients and non-HD CKD stage G5 patients. Age and duration of HD are shown as means ± SD.

	HD (n = 11)	Non-HD CKD Stage 5 (n = 13)
Gender (female)	6 (55%)	9 (69%)
Age (years)	61.8 ± 6.3	59.5 ± 14.0
Duration of HD (years)	8.1 ± 8.2	Not Applicable
Underlying renal disease		
Non-diabetes	7 (64%)	8 (62%)
Diabetes	4 (36%)	5 (38%)
Previous history		
Cardiovascular disease	3 (27%)	3 (23%)
Medication		
Anti-hypertensive agents	6 (55%)	13 (100%)
Statins	0 (0%)	2 (15%)

**Table 2:** Classification of the solutes based on their correlations with estimated glomerular filtration rate (eGFR) in our previous study [8].

		Concentrations in HD Patients Compared to CKD Stage G5 Patients	
		Higher	Lower
Correlations with eGFR in non-HD CKD patients [8]	Negative (Uremic)	**Group A: Uremic solutes more accumulated in HD patients (26 solutes)** Adipate *, Malonate *, ADMA *, Phthalate, *N*-Acetylglutamate, Gluconate, *trans*-Aconitate, Pimelate, Hippurate, 2-Isopropylmalate, *N*-Acetylaspartate, Glutarate, Citramalate, *cis*-Aconitate, 3-Indoxyl sulfate, Isethionate, Ophthalmate, Trimetylamine *N*-oxide, Creatininte, 7-Methylguanine, *N*-Acetylglucosamine, Indole-3-acetate, Cytosine, γ-Butyrobetaine, *N*-ε-Acetyllysine, 1-Methyladenosine	**Group D: Uremic solutes less accumulated in HD patients (8 solutes)** *N*-Acetyl-β-alanine, Succinate, Citrate, SDMA, Methionine sulfoxide, Oxamate †, 4-Hydroxy-3-methoxybenzoate †, Allantoin †
	No correlation (Not uremic)	**Group B: HD patients-specific uremic solutes (10 solutes)** *N*-Acetylneuraminate *, Benzoate *, Fumarate *, Decanoate *, Glycerophosphorylcholine *, Glycerophosphate, Saccharate, *S*-adenosylhomocysteine divalent, Sarcosine, 5-Oxoproline	**Group E: Solutes specifically lost in HD patients (14 solutes)** Mucate, Pelargonate, Homoarginine/*N6-N6-N6*-Trimethyllysine, *o*-Acetylcarnitine, Lysine, Histidine, Glutamine, Methionine, Heptanoate †, 2-Hydroxyoctanoate †, Pentanoate/3-Methylbutanoate †, Piperazine †, 5-Methyltetrahydrofolate †, Butanoate †
	Positive (Not uremic)	**Group C: Solutes decreased less significantly in HD patients (2 solutes)** Tyrosine *, Glutamate *	**Group F: Solute decreased more significantly in HD patients (1 solute)** Lactate

*: Solutes whose serum levels were higher in both pre- and post-HD than in CKD stage G5; †: Solutes that were not detected in either pre- or post-HD.

## Data Availability

Data are available upon request, please contact the contributing author.

## Supplementary Materials

The following are available online at https://www.mdpi.com/article/10.3390/toxins13050324/s1, Figure S1: The 22 anionic solutes whose concentrations were about the same between pre-HD and CKD stage G5, Figure S2: The 39 cationic solutes whose concentrations were about the same between pre-HD and CKD stage G5, Table S1: The complete data on serum concentrations of 54 anionic solutes, Table S2: The complete data on serum concentrations of 68 cationic solutes.
