# Supplementary material for: CE-MS-Based Identification of Uremic Solutes Specific to Hemodialysis Patients
Source: Toxins (Basel). 2021 Apr 30;13(5):324. doi: 10.3390/toxins13050324 (PMC8147146; doi:10.3390/toxins13050324)
Supplement: Supplementary file 1 [file toxins-13-00324-s001.zip › toxins-1180816 SPM.pdf]

# Supplementary Materials: CE-MS-Based Identification of Uremic Solutes Specific to Hemodialysis Patients

**Yasutoshi Akiyama, Koichi Kikuchi, Takafumi Toyohara, Eikan Mishima, Chitose Suzuki, Takehiro Suzuki, Masaaki Nakayama, Yoshihisa Tomioka, Tomoyoshi Soga and Takaaki Abe**

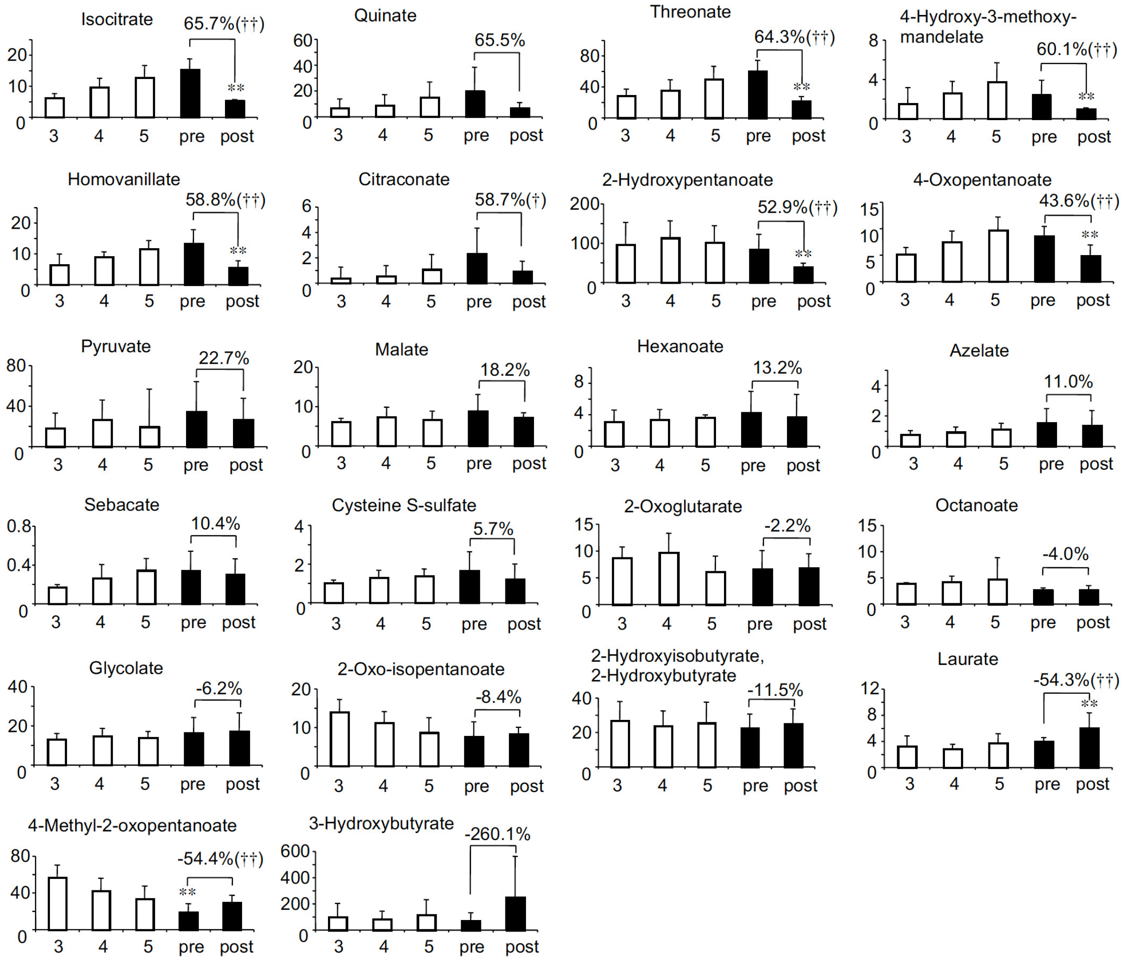


**Figure S1.** The 22 anionic solutes whose concentrations (μM) were about the same between pre- hemodialysis (HD) and chronic kidney disease (CKD) stage G5. Percent values indicate removal rates. CKD stage G3, stage G4, stage G5, pre-HD and post-HD are abbreviated as 3, 4, 5, pre and post, respectively. † $p < 0.05$ between pre- and post-HD, †† $p < 0.01$ between pre- and post-HD. ** $p < 0.01$ VS. CKD stage G5.

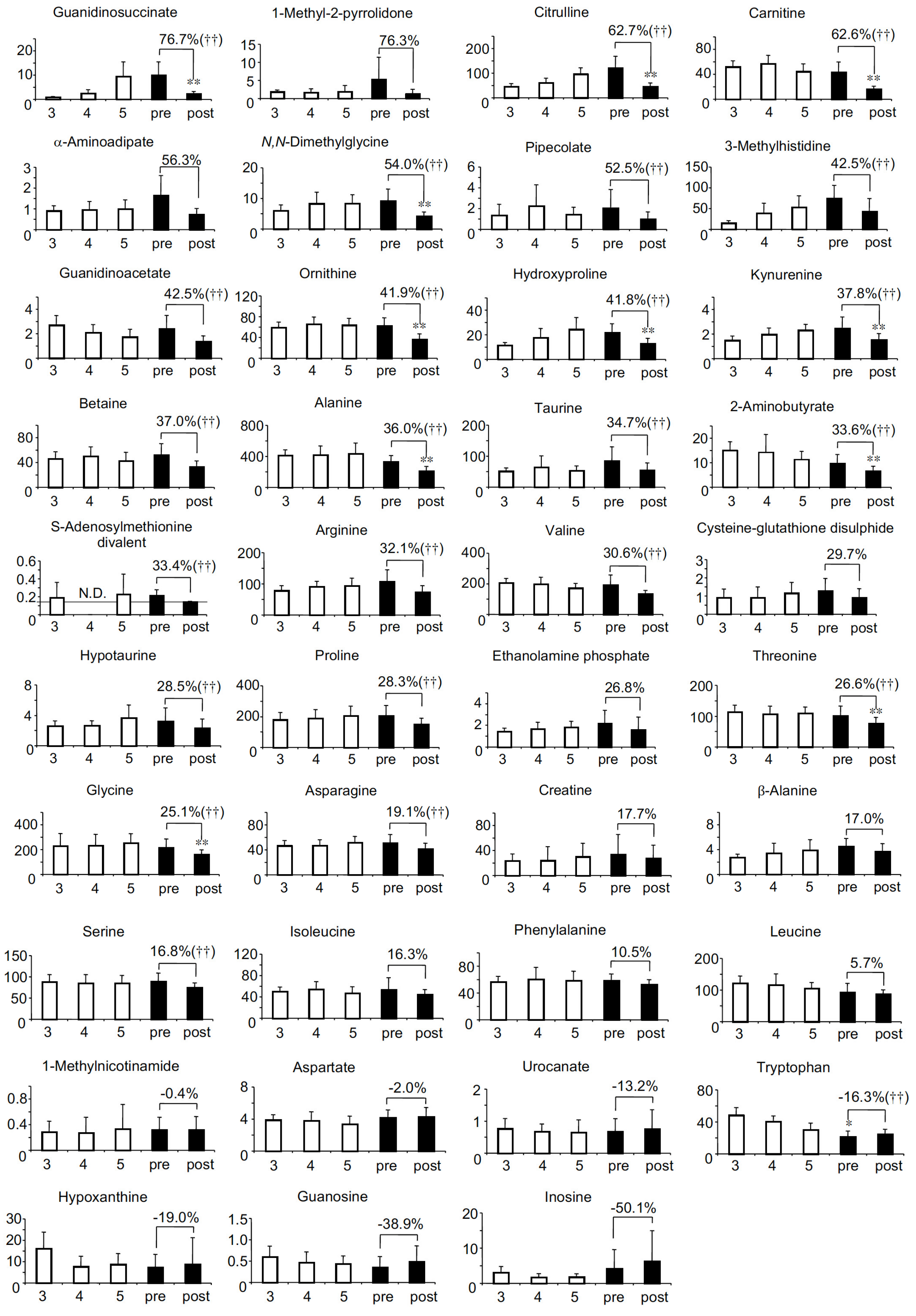


**Figure S2.** The 39 cationic solutes whose concentrations (µM) were about the same between pre-HD and CKD stage G5. Percent values indicate removal rates. N.D.: not detected. CKD stage G3, stage G4, stage G5, pre-HD and post-HD are abbreviated as 3, 4, 5, pre and post, respectively. †† $p < 0.01$ between pre- and post-HD. ** $p < 0.01$ VS. CKD stage G5. Gray line represents the detection limit.
